# Predictors of renal recovery in patients with pre-orthotopic liver transplant (OLT) renal dysfunction

**DOI:** 10.1186/1471-2369-14-147

**Published:** 2013-07-13

**Authors:** Jose Iglesias, Elliot Frank, Sushil Mehandru, John M Davis, Jerrold S Levine

**Affiliations:** 1 Department Medicine subsection of Nephrology, UMDNJ School of Osteopathic Medicine, Stratford, NJ 08084, USA; 2 Department of Medicine subsection Nephrology, Jersey Shore University Medical Center Neptune, Neptune, NJ, USA; 3 Robert Wood Johnson School of Medicine, New Brunswick, NJ, USA; 4 Department of Medicine, Jersey Shore University Medical Center, Neptune, NJ, USA; 5 Department of Surgery, Jersey Shore University Medical Center, Neptune, NJ, USA; 6 Department of Medicine, Section of Nephrology, University of Illinois at Chicago, Chicago, IL 60612, USA; 7 Jesse Brown Veterans Affairs Medical Center, Chicago, IL 60612, USA

## Abstract

**Background:**

Renal dysfunction occurs commonly in patients awaiting orthotopic liver transplantation (OLT) for end-stage liver disease. The use of simultaneous liver-kidney transplantation has increased in the MELD scoring era. As patients may recover renal function after OLT, identifying factors predictive of renal recovery is a critical issue, especially given the scarcity of available organs.

**Methods:**

Employing the UNOS database, we sought to identify donor- and patient-related predictors of renal recovery among 1720 patients with pre-OLT renal dysfunction and transplanted from 1989 to 2005. Recovery of renal function post-OLT was defined as a composite endpoint of serum creatinine (SCr) ≤1.5 mg/dL at discharge and survival ≥29 days. Pre-OLT renal dysfunction was defined as any of the following: SCr ≥2 mg/dL at any time while awaiting OLT or need for renal replacement therapy (RRT) at the time of registration and/or OLT.

**Results:**

Independent predictors of recovery of renal function post-OLT were absence of hepatic allograft dysfunction, transplantation during MELD era, recipient female sex, decreased donor age, decreased recipient ALT at time of OLT, decreased recipient body mass index at registration, use of anti-thymocyte globulin as induction therapy, and longer wait time from registration. Contrary to popular belief, a requirement for RRT, even for prolonged periods in excess of 8 weeks, was not an independent predictor of failure to recover renal function post-OLT.

**Conclusion:**

These data indicate that the duration of renal dysfunction, even among those requiring RRT, is a poor way to discriminate reversible from irreversible renal dysfunction.

## Background

Renal dysfunction occurs commonly in patients with end-stage liver disease (ESLD) awaiting orthotopic liver transplantation (OLT) [1,2]. In the MELD (Model End-Stage Liver Disease) scoring era, the use of simultaneous liver-kidney transplantation (SLKT) has increased [3,4]. From a renal standpoint, considerable uncertainty remains as to which patients will benefit from SLKT [4]. In many cases, pre-OLT renal dysfunction is ameliorated by OLT [5,6]. Moreover, in patients with ESLD and renal dysfunction, studies evaluating factors predictive of recovery of renal function and/or or risk factors predictive of non-recovery of renal function post-OLT have yielded conflicting results [4,7].

Several factors complicate the decision whether patients should receive SLKT. Most important is the complex interaction between the liver and kidney. The cirrhotic milieu often leads to dramatic reductions in renal perfusion, so that renal dysfunction in the setting of cirrhosis may reflect prerenal factors rather than intrinsic renal damage. In the case of prerenal dysfunction, OLT should lead to nearly full recovery of renal function. While a renal biopsy can easily distinguish between prerenal and intrinsic causes for renal insufficiency, the coagulopathy associated with cirrhosis frequently precludes performing a biopsy [1,8-10]. These issues are highlighted by the paucity of pathological data in nearly all studies of the hepatorenal syndrome (HRS) [11,12].

Given the scarcity of available donor renal allografts, identifying which patients with pre-OLT renal dysfunction will regain renal function post-OLT becomes an even more critical issue. Previous studies have been hampered by small population sizes, single center experiences, and the retrospective nature of the data [8,10]. Employing the UNOS database, we sought to identify donor- and patient-related predictors of renal recovery and non-recovery in 1720 patients with pre-OLT renal dysfunction and transplanted from 1989 to 2005. In this cohort, the most important independent predictor of recovery of renal function post-OLT was an absence of hepatic allograft dysfunction. Other predictors of renal recovery were related to amelioration or reversal of the cirrhotic milieu and/or improved renal perfusion, as reflected by decreased severity of recipient liver disease and a lack of use of calcineurin inhibitors, respectively. An intriguing finding of our study is the potentially beneficial effect of anti-thymocyte globulin induction, which emerged as an independent predictor of renal recovery distinct from its calcineurin inhibitor-sparing effects. Finally, contrary to popular belief, our data suggest that the requirement for renal replacement therapy, even for prolonged periods in excess of 8 weeks, may not be an independent predictor of the failure to recover renal function post-OLT.

## Methods

### Ethics statement

The study was undertaken in accordance with principles of the Declaration of Helsinki and the standards of good clinical practice. The study was approved by the Institutional Review Board and Ethics Committee (IRB#06-008) of Barnabas Health Community Medical Center.

### Study population

The current study was designed as a retrospective cohort study. Data were obtained the United Network for Organ Sharing (UNOS) for donors and OLT recipients transplanted from January, 1989, through March, 2005, the time of creation of our database. Recovery of renal function following OLT was defined as a primary composite end point of a serum creatinine (SCr) ≤1.5 mg/dL at discharge and survival of ≥29 days. Patients included in our analysis were those who possessed pre-OLT renal dysfunction, defined as any of the following: (1) SCr ≥2 mg/dL at any time while awaiting OLT; (2) need for renal replacement therapy (RRT) at the time of registration (dialysis at time of registration); (3) need for RRT at time of OLT (dialysis at time of transplant); (4) need for RRT at time of both registration and OLT (dialysis-dependence). Patients were excluded from analysis if they received multi-organ transplantation, were <17 years old at the time of OLT, or received a living-related or living-unrelated OLT. In addition, patients were excluded if their SCr was ≤1.5 mg/dL at the time of OLT and they had received RRT neither at the time of registration nor at the time of OLT. Data on the type of RRT, whether intermittent or continuous, were not available.

### Data collection

Data collected for OLT recipients included the following: routine demographic variables (age, sex, race); wait time from registration; registration and/or peri-transplant severity of illness characteristics (body mass index [BMI]; intensive care unit [ICU] admission; presence of ascites, hepatic encephalopathy, or portal vein thrombosis [PVT]; need for mechanical ventilation, transjugular intrahepatic portosystemic shunt [TIPS], or RRT); comorbidities (coronary artery disease, diabetes mellitus, hypertension, peripheral arterial disease, cachexia); etiology of ESLD (fulminant hepatic failure, hepatitis B, hepatitis C, non-alcoholic chronic steatohepatitis [NASH], alcoholic cirrhosis, or other); registration and/or peri-transplant physiologic variables (clotting times, serum albumin, liver enzymes, total bilirubin); MELD score; cold and warm ischemia times; occurrence of allograft dysfunction; and immunosuppressive medications used for induction and/or maintenance therapy (antithymocyte globulin, OKT3, basiliximab, daclizumab, alemtuzumab, cyclosporine, tacrilimus, sirolimus). Immunosuppressive usage was reported for the immediate post-OLT period, before recovery of renal function could occur. Renal variables collected for OLT recipients included the requirement for RRT, SCr, and estimated glomerular filtration rate (eGFR) at the times of registration, OLT, and discharge. For patients receiving RRT, several values of SCr were used, an unadjusted and two adjusted values. The unadjusted SCr was the value entered into the UNOS database. Given uncertainty whether the entered SCr was obtained pre- or post-dialysis, analysis was repeated using a single adjusted value for SCr of either 4.0 or 5.0 mg/dL. The former value is in accord with previous work by Bahirwani et al.[13] eGFR was determined using the 4-variable equation of the Modification of Diet in Renal Disease (MDRD) study group. Data collected for donors included age, sex, race, BMI, terminal liver enzymes, terminal total bilirubin, and whether the OLT derived from a non-heart beating donor. Transplant related data include type of induction therapy, initial immunosuppression, cold ischemia time, warm ischemia time and liver allograft function.

### Data analysis

All statistical analyses unless otherwise indicated were performed as a comparison for OLT recipients who recovered renal function vs. those who did not. Summary statistics were computed for the two cohorts, those with recovery of renal function and those with non-recovery. Continuous variables were expressed as mean ± standard deviation and compared by the Student t test or the Wilcoxon rank-sum test. For purposes of statistical analysis, patients receiving RRT were assigned a SCr of 4 mg/dL. Categorical variables were compared by Fisher's exact test or chi-square analysis. Comparison of cohorts included both univariate and multivariate analyses. Candidate variables for multivariate analysis included all variables found to be significantly different by univariate analysis at p < 0.05. To determine variables independently predictive of renal recovery, we performed logistic regression analysis with forward variable selection. Stepwise selections for logistic regression were based on the maximum likelihood ratio. For continuous variables, the odds ratio (OR) represents the relative amount by which the probability of observing recovery of renal function increases or decreases if the independent variable is increased by exactly one unit. OR and their 95% confidence intervals (CI) were determined by exponentiation of the regression coefficient or its upper and lower 95% CI, respectively.

## Results

### Univariate analyses of factors associated with non-recovery of renal function post-OLT within the entire cohort

To determine factors associated with recovery vs. non-recovery of renal function following OLT, we analyzed multiple routinely available demographic, clinical, and laboratory variables obtained in OLT recipients with pre-OLT renal dysfunction who were transplanted between 1989 and 2005.

**Figure 1:**
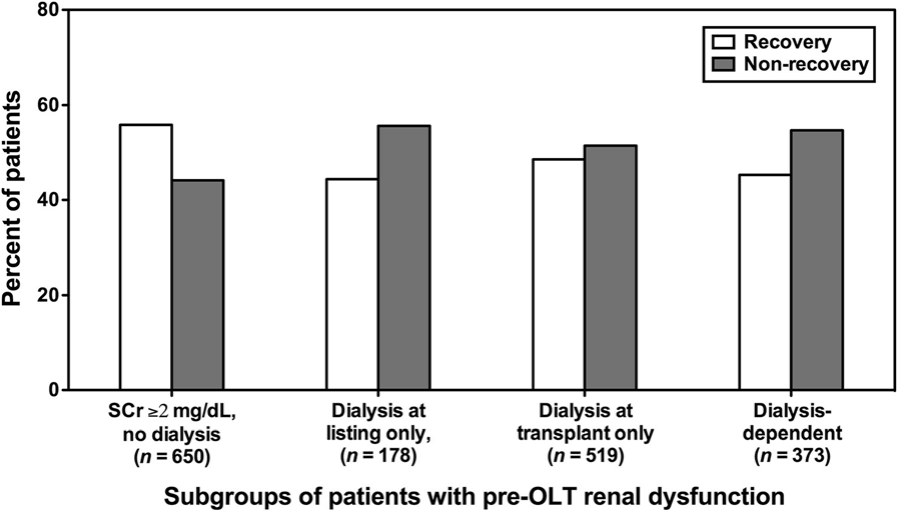
**Recovery versus non**-**recovery of renal function following orthotopic liver transplantation (OLT) in patients with pre-OLT renal dysfunction grouped according to their need for renal replacement therapy (RRT).** Recovery of renal function following OLT was defined as a composite end point of a serum creatinine (SCr) ≤1.5 mg/dL at discharge and survival of ≥29 days. Patients included in our analysis were those who possessed pre-OLT renal dysfunction, defined as any of the following: (1) SCr ≥2 mg/dL at any time while awaiting OLT, but not requiring RRT (SCr ≥2 mg/dL, no dialysis); (2) need for RRT at registration, but not at OLT (dialysis at registration only); (3) need for RRT at OLT, but not at registration (dialysis at transplant only); (4) need for RRT at both registration and OLT (dialysis-dependent).

A total of 1720 patients were evaluated. 863 (51%) patients recovered renal function, while 857 (49%) failed to recover renal function. Notably, of those who recovered renal function, 500 (58%) received RRT at some time prior to OLT. Of these, 79 (16%) were on dialysis only at the time of registration, 252 (50%) were on dialysis only at the time of OLT, and 169 (34%) required RRT throughout their time on the waiting list. A similar distribution was seen for those who failed to recover renal function. 570 (67%) required RRT at some time prior to OLT. 99 (17%) were on dialysis only at the time of registration, 267 (47%) were on dialysis only at the time of transplant, and 204 (36%) required RRT throughout their time on the waiting list. Figure 1 depicts the percentage of patients with recovery vs. non-recovery of renal function according to their need for RRT at the time of registration and/or OLT.

Using our entire cohort (n = 1720), we compared OLT recipients with recovery vs. non-recovery of renal function by univariate analysis for multiple characteristics, including the following: recipient demographic variables and clinical characteristics at both the time of registration and the time of transplant (Table 1); recipient co-morbidities (Table 2); etiology of ESLD (Table 3); common laboratory chemistries (Table 4); MELD score and allograft-related factors (Table 5); immunosuppressive regimen (Table 6); and donor demographic variables and clinical features (Table 7).

**Table 1:** **Demographic**, **listing**, **and peri**-**transplant characteristics in patients with pre**-**OLT renal dysfunction that are associated with recovery of renal function post**-**OLT**

	**Recovery of renal function (n=863)**	**Non-recovery (n=857)**	**p**	**OR (95% CI)**
**Demographic characteristics**				
Age	50.3 ± 10.2	50.0 ± 10.5	0.3	
Sex (male)	544 (63%)	581 (67%)	0.043	0.81 (0.66-0.98)
Race (Caucasian)	599 (70%)	620 (72%)	0.21	0.88 (0.70-1.07)
**Listing characteristics**				
ICU admission	207 (24%)	255 (29%)	0.007	0.74 (0.60-0.92)
Mechanical ventilation	108 (13%)	172 (20%)	0.00001	0.57 (0.43-0.74)
Encephalopathy	510 (59%)	541 (60%)	0.35	1.08 (0.9-1.3)
Ascites	593 (72%)	598 (63%)	0.60	0.95 (0.75-1.16)
TIPS	57 (6.6%)	63 (7%)	0.63	0.92 (0.65-1.30)
PVT	17 (1.9%)	30 (3.5%)	0.052	0.55 (0.32-0.93)
Dialysis (at listing)*	248 (28%)	303 (32%)	0.003	0.63 (0.50-0.76)
BMI (kg/m^2^)	27.6 ± 5.5	28.4 ± 6.1	0.02	
**Peri-transplant characteristics**				
Wait time from listing (days)	178 ± 297	145 ± 266	0.00001	
ICU admission	441 (51%)	417 (49%)	0.76	1.03 (0.85-1.20
Mechanical ventilation	206 (24%)	249 (29%)	0.015	0.76 (0.62-0.95)
Encephalopathy	343 40%)	344 (41%)	0.86	0.98 (0.81-1.20)
Ascites	580 (67%)	547 (64%)	0.14	1.20 (0.95-1.40)
TIPS	75 (8.6%)	79 (9.2%)	0.70	0.93 (0.67-1.30)
PVT	22 (2.5%)	39 (4.5%)	0.025	0.55 (0.32-0.99)
Dialysis (at transplant)*	421 (49%)	471 (55%)	0.01	0.70 (0.57-0.85)
Dialysis (at both listing and transplant)	169 (19%)	204 (23%)	0.03	0.78 (0.62-0.99)
BMI (kg/m^2^)	27.0 ± 6.4	28.0 ± 6.3	0.019	

Abbreviations: *BMI* body mass index, *CI* confidence interval, *ICU* intensive care unit, *OLT* orthotopic liver transplant, *OR* odds ratio, *PVT* portal vein thrombosis, *TIPS* transjugular intrahepatic porto-systemic shunt.

* The number of patients on dialysis at listing (n = 551) includes those on dialysis only at listing (n = 178) as well as those on dialysis at both listing and transplant (n = 373). Similarly, the number of patients on dialysis at transplant (n = 892) includes those on dialysis only at listing (n = 519) as well as those on dialysis at both listing and transplant (n = 373).

**Table 2:** **Co**-**morbidities in patients with pre**-**OLT renal dysfunction associated with recovery of renal function post**-**OLT**

	**Recovery of renal function(n = 863)**	**Non-recovery (n = 857)**	**p**	**OR (95% CI)**
Coronary artery disease	9 (1%)	12 (1.3%)	0.66	0.68 (0.30-1.50)
Diabetes mellitus	204 (24%)	235 (27%)	0.07	0.84 (0.70-1.00)
Hypertension	158 (18%)	167 (19%)	0.53	0.90 (0.72-1.17)
Peripheral arterial disease	7 (0.8%)	9 (1%)	0.56	0.74 (0.27-2.00)
Chronic obstructive pulmonary disease	4 (0.5%)	11 (1.3%)	0.07	0.38 (0.14-1.12)
Re-transplantation	136 (16%)	178 (21%)	0.007	0.70 (0.56-0.90)
Variceal bleeding	66 (7.7%)	91 (11%)	0.032	0.70 (0.50-0.97)
Cachexia	218 (25%)	228 (26%)	0.52	0.93 (0.75-1.15)

Abbreviations: *CI* confidence interval, *OLT* orthotopic liver transplantation, *OR* odds ratio.

**Table 3:** **Etiology of end**-**stage liver disease in patients with pre**-**OLT renal dysfunction associated with recovery of renal function post**-**OLT**

	**Recovery of renal function (n = 863)**	**Non-recovery (n = 857)**	**p**	**OR (95% CI)**
Fulminant hepatic failure	89 (10%)	118 (14%)	0.039	0.73 (0.54-0.98)
Hepatitis B	41 (5.4%)	33 (4%)	0.27	1.29 (0.80-1.50)
Other	300 (36%)	318 (38%)	0.62	0.90 (0.77-1.10)
NASH	4 (0.4%)	12 (1.4%)	0.04	0.32 (0.1-1.0)
Hepatitis C	246 (28%)	223 (26%)	0.56	1.06 (0.97-1.30)
Alcoholic cirrhosis	183 (21%)	155 (18%)	0.10	1.20 (0.96-1.50)

Abbreviations: *CI* confidence interval, *NASH* non-alcoholic steatohepatitis, *OLT* orthotopic liver transplant, *OR* odds ratio.

**Table 4:** **Laboratory values in patients with pre**-**OLT renal dysfunction associated with recovery of renal function post**-**OLT**

	**Recovery of renal function(n = 863)**	**Non-recovery (n = 857)**	**p**
SCr at time of registration (mg/dL)*	2.4 ± 2.0	2.9 ± 2.6	0.00001
SCr at time of registration (mg/dL)			
(adjusted for dialysis, SCr = 4 mg/dL)*	2.5 ± 1.6	2.4 ± 1.7	0.23
SCr at time of registration (mg/dL)			
(adjusted for dialysis, SCr = 5 mg/dL)*	2.8 ± 2.0	2.78 ± 2.0	0.30
SCr at time of transplant	3.6 ± 2.0	4.0 ± 2.3	0.00001
SCr at time of transplant (mg/dL)			
(adjusted for dialysis, SCr = 4 mg/dL)	3.84 ± 1.5	3.8 ± 1.4	0.52
SCr at time of transplant (mg/dL)			
(adjusted for dialysis, SCr = 5 mg/dL)	4.3 ± 1.6	4.3 ± 1.6	0.80
eGFR at time of registration (cc/min)	57 ± 41	50 ± 42	0.00001
eGFR at time of registration (cc/min)			
(adjusted for dialysis, SCr = 4 mg/dL)	51 ± 42	53 ± 44	0.33
eGFR at time of registration (cc/min)			
(adjusted for dialysis SCr = 5 mg/dL)	51 ± 43	53 ± 46	0.33
eGFR at time of transplant (cc/min)	28 ± 21	22 ± 14	0.00001
eGFR at time of transplant (cc/min)			
(adjusted for dialysis, SCr = 4 mg/dL)	20 ± 14	21 ± 12	0.30
eGFR at time of transplant (cc/min)			
(adjusted for dialysis, SCr = 5 mg)	19 ± 14	19 ± 12	0.49
Serum ALT at time of transplant (units/L)	257 ± 888	489 ± 1379	0.007
INR at time of transplant	2.1 ± 1.5	2.2 ± 2.8	0.80
Total bilirubin at time of transplant (mg/dL)	14.0 ± 14.4	16.0 ± 16.0	0.38
Serum albumin at time of transplant (g/dL)	2.78 ± 0.76	2.82 ± 0.77	0.18

* For patients receiving RRT, several values of SCr were used, an unadjusted and two adjusted values. The unadjusted SCr was the value entered into the UNOS database. Given uncertainty whether the entered SCr was obtained pre- or post-dialysis, analysis was repeated using a single adjusted value for SCr of either 4.0 or 5.0 mg/dL.

Abbreviations: *ALT* alanine transaminase, *eGFR* estimated glomerular filtration rate, *INR* international normalized ratio, *OLT* orthotopic liver transplantation, *SCr* serum creatinine.

**Table 5:** **Allograft**-**associated characteristics of patients with pre**-**OLT renal dysfunction associated with recovery of renal function post**-**OLT**

	**Recovery of renal function (n = 863)**	**Non-recovery (n = 857)**	**p**	**OR (95% CI)**
Transplanted prior to MELD era	275 (31%)	344 (40%)	0.0001	0.69 (0.57-0.85)
Whole organ transplant	846 (98%)	846 (99%)	0.23	0.65 (0.31-1.21)
MELD score	33.7 ± 8.7	34 ± 9.2	0.40	
Cold ischemia time (hrs)	7.6 ± 3.5	7.7 ± 3.6	0.98	
Warm ischemia time (mins)	42 ± 20	41 ± 18	0.51	
Allograft dysfunction	111 (13%)	303 (35%)	0.00001	0.27 (0.21-0.34)

Abbreviations: *CI* confidence interval, *MELD* model end-stage liver disease, *OLT* orthotopic liver transplantation, *OR* odds ratio.

**Table 6:** **Immunosuppression in patients with pre**-**OLT renal dysfunction associated with recovery of renal function post**-**OLT**

	**Recovery of renal function (n = 863)**	**Non-recovery (n = 857)**	**p**	**OR (95% CI)**
Anti-thymocyte globulin induction	34 (4%)	16 (1.8%)	0.014	2.15 (1.2-4.0)
OKT3	20 (2.3%)	20 (2.3%)	0.90	0.99 (0.53-1.73)
Basiliximab	26 (3%)	32 (3.7%)	0.69	0.80 (0.55-1.50)
Daclizumab	20 (2.3%)	31 (3.6%)	0.27	0.74 (0.43-1.27)
Alemtuzumab	1 (0.1%)	2 (0.2%)	0.54	0.50 (0.44-5.30)
Cyclosporine	188 (22%)	192 (22%)	0.75	0.96 (0.76-1.21)
Tacrolimus	437 (51%)	479 (56%)	0.029	0.81 (0.67-0.97)
Sirolimus	21 (3%)	23 (2.7%)	0.74	0.87 (0.81-1.23)

Abbreviations: *CI* confidence interval, *OLT* orthotopic liver transplant, *OR* odds ratio.

**Table 7:** **Donor characteristics in patients with pre**-**OLT renal dysfunction associated with recovery of renal function post**-**OLT**

	**Recovery of renal function (n = 863)**	**Non-recovery (n = 857)**	**p**	**OR (95% CI)**
Donor age	37 ± 17	39 ± 17	0.00001	
Donor sex (male)	542 (62%)	536 (62)%	1.0	1.01 (0.87-1.19)
Donor race (Caucasian)	611 (71%)	603 (70%)	0.9	0.94 (0.97-1.12)
Donor BMI	25.6 ± 6.1	27.5 ± 13.0	0.007	
Non-heart-beating donor	14 (1.6%)	14 (1.6%)	0.95	1.00 (0.47-1.80)
Donor ALT	53 ± 95	59 ± 158	0.12	
Donor AST	70 ± 96	75 ± 176	0.35	
Donor bilirubin (total)	1.0 ± 1.8	1.0 ± 1.7	0.79	

Abbreviations: *ALT* alanine transaminase, *AST* asparatate transaminase, *BMI* body mass index, *CI* confidence interval, *OLT* orthotopic liver transplant, OR odds ratio.

The following recipient-related demographic and clinical characteristics were associated with recovery of renal function post-OLT (Table 1): female sex (p < 0.05); lack of ICU admission at the time of registration (p = 0.007); lack of need for mechanical ventilation either at the time of registration (p = 0.00001) or at the time of OLT (p = 0.015); absence of PVT at the time of OLT (p = 0.025); lack of need for RRT at the time of registration (p = 0.003), at the time of OLT (p = 0.01), or at both times (p = 0.03); a longer time on the waiting list (p = 0.00001); and a lower BMI either at the time of registration (p = 0.019) or at the time of OLT (p = 0.02).

OLT recipients who recovered renal function were more likely to be receiving their first OLT (p = 0.007) and to have no history of variceal bleeding (p = 0.032) (Table 2). The etiology of their ESLD was less likely to be fulminant hepatic failure (p = 0.039) or NASH (p = 0.04) (Table 3). They were also less likely to have an elevated alanine transaminase (ALT) (p = 0.007) (Table 4).

Recovery of renal function was associated with an absence of allograft dysfunction in the immediate post-OLT period (p = 0.00001) and with transplantation during the MELD era (p = 0.0001) (Table 5). Aspects of the immunosuppressive regimen also correlated with the likelihood of renal recovery. Recovery of renal function was associated with use of antithymocyte globulin as an induction agent (p = 0.014) and absence of use of tacrolimus (p = 0.029) (Table 6).

With respect to donor-related factors (Table 7), recovery of renal function occurred more frequently in recipients whose allografts came from younger donors (p = 0.00001) and donors with a smaller BMI (p = 0.007).

Finally, OLT recipients who recovered renal function post-OLT had lower SCr and higher eGFR both at the time of registration (p = 0.00001) and at the time of transplant (p = 0.00001). It is important, however, to note that these analyses included patients undergoing RRT, for whom measurement of SCr bore an uncertain temporal relationship to their dialysis. When parameters of renal function were adjusted for patients receiving RRT by assigning them a uniform SCr value of either 4.0 mg/dL or 5.0 mg/dL, consistent with minimal renal function, then neither SCr or eGFR retained its correlation with non-recovery of renal function (Table 4). We return to this important issue later (see Tables 8 and 9, and Discussion).

**Table 8:** **Multivariate analysis of factors independently associated with recovery of renal function post**-**OLT in all patients with pre**-**OLT renal dysfunction**

	**Regression coefficient**	**S.E.**	**p**	**OR (95% CI)**	Coefficient of determination*
Allograft dysfunction	−1.40	0.11	0.00001	0.25 (0.18-0.33)	0.047
Transplanted before MELD era	−0.363	0.12	0.02	0.69 (0.55-0.87)	0.010
Sex (male)	−0.416	0.12	0.00001	0.66 (0.53-0.83)	0.009
Donor age	−0.008	0.003	0.014	0.992 (0.986-0.999)	0.007
Recipient ALT at time of transplant	−0.0001	0.0001	0.026	1.0005 (1.000-1.001)	0.007
Recipient BMI at time of listing	−0.027	0.009	0.005	0.974 (0.95-0.99)	0.005
Anti-thymocyte globulin induction	0.79	0.34	0.022	2.2 (1.12-4.30)	0.004
Wait time from listing (days)	0.0001	0.152	0.026	1.000 (1.000-1.001)	0.004

Abbreviations: *ALT* alanine transaminase, *BMI* body mass index, *CI* confidence interval, *MELD* model end-stage liver disease, *OLT* orthotopic liver transplantation, *OR* odds ratio.

* The cumulative coefficient of determination for all variables combined is 0.093.

**Table 9:** **Multivariate analysis of factors independently associated with recovery of renal function post**-**OLT stratified according to groups**

	**Regression coefficient**	**S**.**E**.	**p**	**OR (95% CI)**	**Coefficient of variation**
**SCr ≥2 mg/dL, not requiring dialysis (n = 650)**					
Allograft dysfunction	−1.388	0.090	0.0001	0.061 (0.163-0.383)	0.11
**Dialysis at registration only**					
**(n = 178)**					
Allograft dysfunction	−1.86	0.55	0.00001	0.159 (0.054-0.990)	0.119
MELD score	−0.019	0.021	0.016	0.95 (0.91-0.99)	0.058
**Dialysis at transplant only**					
**(n = 519)**					
Allograft dysfunction	−1.42	0.26	0.00001	0.24 (0.14-0.40)	0.084
Transplanted before MELD	−0.83	0.21	0.00001	0.43 (0.289-0.670)	0.033
Anti-thymocyte globulin induction	1.76	0.70	0.010	4.80 (1.23-19.10)	0.019
Cyclosporine	−0.77	0.27	0.030	0.46 (0.27-0.78)	0.011
Tacrolimus	−0.5	0.23	0.029	0.40 (0.40-0.95)	0.011
**Dialysis-dependence**					
**(n = 373)**					
Allograft dysfunction	−1.752	0.358	0.00001	0.173 (0.086-0.350)	0.139
Donor age	−0.019	0.008	0.020	0.98 (0.96-1.0)	0.044
Recipient bilirubin (total)	−0.025	0.009	0.004	0.97 (0.96-0.99)	0.040
Donor ALT	0.008	0.003	0.020	1.008 (1.001-1.015)	0.028

Abbreviations: *ALT* alanine transaminase, *BMI* body mass index, *CI* confidence interval, *MELD* model end-stage liver disease, *OLT* orthotopic liver transplantation, *OR* odds ratio, *SCr* serum creatinine.

### Multivariate analysis of factors associated with non-recovery of renal function post-OLT within the entire cohort

We used forward stepwise logistic regression analysis to determine which variables identified by univariate analyses (Tables 1, 2, 3, 4, 5, 6 and 7) were independent predictors of recovery of renal function (Table 8). Independent predictors of recovery of renal function post-OLT, in descending order of coefficient of determination, were absence of hepatic allograft dysfunction, transplantation during the MELD era, female sex of the recipient, decreased donor age, decreased recipient ALT at OLT, decreased recipient BMI at registration, use of anti-thymocyte globulin as induction therapy, and a longer wait time from registration.

### Multivariate analysis of factors associated with non-recovery of renal function post-OLT within subgroups stratified according to the need for RRT

To determine whether the predictors of recovery of renal function varied with the level of renal dysfunction prior to OLT, as well as to address uncertainty in the measurement of SCr among patients receiving RRT (see Table 4), we performed a subgroup analysis of our cohort. We stratified the cohort into the following 4 groups: (1) recipients with SCr ≥2 mg/dL at any time while awaiting OLT, but not requiring RRT (n = 650); (2) recipients requiring RRT at the time of registration, but not at the time of transplant (dialysis at registration only, n = 178); (3) recipients requiring RRT at the time of transplant, but not at the time of registration (dialysis at transplant only, n = 519); (4) recipients requiring RRT at both the time of registration and the time of transplant (dialysis-dependence, n = 373).

For each subgroup, we performed a separate forward stepwise logistic regression analysis using variables found to be significant by univariate analysis (data not shown). The results of the multivariate analysis for each subgroup are summarized in Table 9. Notably, an absence of hepatic allograft dysfunction was an independent predictor of recovery of renal function in all 4 subgroups. Among patients with a SCr ≥ 2 mg/dl at any time prior to OLT, but not requiring RRT, an absence of allograft dysfunction was the only independent predictor of recovery. Among patients requiring RRT at registration only, the independent predictors of recovery of renal function, in descending order of coefficient of determination, were absence of hepatic allograft dysfunction and a lower MELD score. Among patients requiring RRT at transplant only, the independent predictors of recovery of renal function, in descending order of coefficient of determination, were an absence of allograft dysfunction, transplantation during the MELD era, use of anti-thymocyte globulin as induction therapy, lack of use of cyclosporine, and lack of use of tacrolimus. The emergence of both anti-thymocyte globulin induction therapy and lack of use of cyclosporine or tacrolimus as independent predictors suggests that the beneficial effect of anti-thymocyte globulin induction extends beyond its sparing of calcineurin inhibitor use. Finally, among patients requiring RRT at both registration and transplant, the independent predictors of recovery of renal function, in descending order of coefficient of determination, were an absence of allograft dysfunction, decreased donor age, decreased recipient total bilirubin, and increased donor ALT. With regard to increased donor ALT, the weakest of the predictors, it is noteworthy that, despite statistical difference donor ALT was within the normal range for patients with recovery vs. non-recovery of renal function.

### Analysis of the effect of time on waiting list on post-OLT recovery of renal function

The duration of renal dysfunction is thought to impact the likelihood of recovery of renal function post-OLT [14]. Even when pre-OLT renal dysfunction is thought to be solely prerenal, prolonged impairment of renal perfusion because of the cirrhotic milieu may lead to slow ischemic dropout of glomeruli and tubules [12]. We therefore analyzed the effect of time on the waiting list among the 4 subgroups of OLT recipients with pre-OLT renal dysfunction.

The mean duration of time on the waiting list did not differ for OLT recipients with recovery vs. non-recovery of renal function for any of the 4 subgroups with pre-OLT renal dysfunction (Table 10). Remarkably, this was true even for those patients who received RRT both at registration and at OLT (p = 0.91). A recent Consensus Conference on SLKT identified 8 weeks as a threshold duration of RRT beyond which the likelihood of renal recovery was sufficiently low as to justify use of SLKT [15]. We therefore repeated our analysis, restricting our comparison to OLT recipients with wait times ≥8 weeks. We again found no difference in the percentage of patients with recovery vs. non-recovery of renal function for OLT recipients whose wait times exceeded 8 weeks (Table 11). Notably, this includes those recipients requiring RRT at both registration and transplant (Table 11). Thus, among 82 dialysis-dependent patients with wait times of ≥8 weeks, 38 (46%) had recovery of renal function and 44 (54%) had non-recovery (OR = 1.05, 95% CI = 0.65-1.75, p = 0.90). In fact, recipients receiving RRT at the time of OLT and with a wait time >8 weeks had an increased likelihood of recovery vs. non-recovery of renal function (Table 11). Finally, we used Fisher's exact test to compare the likelihood of renal recovery in the subgroup of dialysis-dependent patients, stratified according to whether their wait time was ≤8 weeks vs. >8 weeks (Table 12). The percentage of patients having recovery of renal function was the same regardless of wait time (45% for ≤8 weeks vs. 46% for >8 weeks, p = 0.90).

**Table 10:** **Wait time in days from listing until OLT in patients with recovery vs**. **non**-**recovery of renal function post**-**OLT grouped by categories of pre**-**OLT renal dysfunction**

	**Recovery of renal function**	**Non**-**recovery**	**p**
**SCr ≥2 mg/dL, not requiring dialysis (n = 650)**	363 ± 233	287 ± 217	0.51
Dialysis at time of listing (n = 178)	125 ± 224	84 ± 210	0.21
**Dialysis at time of transplant (n = 519)**	251 ± 182	261 ± 146	0.16
**Dialysis-dependence (n = 373)**	79 ± 170	76 ± 180	0.91

**Table 11:** **Percent of patients with wait time greater than 8 weeks stratified according to recovery vs**. **non**-**recovery of renal function post**-**OLT grouped by categories of pre**-**OLT renal dysfunction**

	**Recovery of renal function**	**Non-recovery**	**p**	**OR (95% CI)**
SCr ≥2 mg/dL, not requiring dialysis (n = 391)	219 (56%)	172 (44%)	0.9	1.01 (0.84-1.20)
Dialysis at time of listing (n = 51)	28 (55%)	23 (45%)	0.73	1.90 (0.94-3.50)
Dialysis at time of transplant (n = 211)	114 (54%)	97 (46%)	0.035	1.45 (1.026-2.07)
Dialysis-dependence (n = 82)	38 (46%)	44 (54%)	0.9	1.05 (0.65-1.75)

**Table 12:** **Recovery of renal function among dialysis**-**dependent recipients of OLT stratified according to wait time greater than or less than 8 weeks**

	**Recovery of renal function(n = 169)**	**Non**-**recovery (n = 204)**	**p**	**OR (95% CI)**
Wait time >8 weeks	38 (46%)	44 (54%)	0.90	1.05 (0.65-1.75)
(n = 82)				
Wait time ≤8 weeks	131 (45%)	160 (55%)	0.87	1.025 (0.80-1.30)
(n = 291)				

Abbreviations: *CI* confidence interval, *OLT* orthotopic liver transplantation, *OR* odds ratio, *SCr* serum creatinine.

## Discussion

Using the UNOS database, we performed a retrospective analysis to determine factors predictive of recovery of renal function among OLT recipients with pre-OLT renal dysfunction. We defined recovery of renal function as a composite end point of SCr ≤1.5 mg/dL at discharge and survival ≥29 days. We report several major findings.

First, as in previous studies, recovery of renal function occurred in ~50% of patients with pre-OLT renal dysfunction. This percentage was consistent across all severities of pre-OLT renal function, including patients who required RRT at registration and/or OLT (cf. Figure 1). In accord with our data, Marik et al. reported that 58% of 28 patients with type I HRS recovered renal function post OLT [6]. Shusterman et al. described post-OLT recovery of renal function in 13 of 17 patients (77%) with pre-OLT HRS (77%) and in 8 of 12 (67%) with pre-OLT acute kidney injury (AKI) [16]. Davis et al. reported that only 1.5% of patients with severe pre-OLT renal dysfunction (eGFR <30 ml/min) required kidney transplantation within 1 year after OLT, suggesting that recovery of renal function had occurred in a large percentage of these patients [4]. Notably, in the MELD era, for patients requiring RRT at the time of registration, ~15% of those listed for OLT alone and ~6.5% of those listed for SKLT no longer required RRT at the time of OLT [4]. Northrup et al., evaluating 1041 patients with pre-OLT renal dysfunction requiring RRT from 2002 to 2007, observed spontaneous renal recovery in 68% of patients [5]. Finally, in a cohort of 155 patients who received SKLT, Levitsky et al. observed that up to 40% of patients recovered function of their native kidneys, with post-OLT GFR of their native kidneys being in the range of GFR 30 to 40 mL/min, as assessed by nuclear scanning [17]. Thus, across a broad range of studies, recovery of renal function is a not uncommon occurrence among OLT recipients with pre-OLT renal dysfunction, even those requiring RRT.

Second, the single factor most predictive of renal recovery was an absence of hepatic allograft dysfunction. This was true across all levels of pre-OLT renal dysfunction. Thus, an absence of hepatic allograft dysfunction emerged as the strongest independent predictor of renal recovery not only in our entire cohort of 1720 patients (Table 8), but also in each of our four subgroups of renal dysfunction (Table 9): (1) SCr ≥2 mg/dL at any time pre-OLT, but not requiring RRT (n = 650); (2) RRT at registration, but not at OLT (n = 178); (3) RRT at OLT, but not at registration (n = 519); (4) RRT at both registration and OLT (n = 373). Remarkably, in the first subgroup, an absence of allograft dysfunction was the only independent predictor of renal recovery, whereas in the second subgroup it was the stronger of only two independent predictors.

We speculate that hepatic allograft dysfunction may impede recovery of renal function through multiple mechanisms. Possible contributory factors include persistence of the cirrhotic milieu, surgical complications, need for re-operation, and adverse systemic factors associated with allograft dysfunction, such as vascular thrombosis, reperfusion syndrome, hemorrhage, inflammation, or infection [18,19]. Such factors may not only prevent recovery of renal function post-OLT, but also lead to superimposed AKI. Indeed, several studies have observed a strong association between hepatic allograft dysfunction and post-OLT renal dysfunction and/or need for RRT [19,20].

Other predictors of post-OLT recovery of renal function, identified within our entire cohort or within one or more of our subgroups, may also relate indirectly to allograft dysfunction. For example, our study concurs with others in showing a worse outcome with the use of older donors [5,21,22] (Tables 8 and 9). While the mechanism for the detrimental effect of older donor age on renal recovery is not fully understood, a slower onset of hepatic allograft function may be contributory [23,24]. There is also evidence that the livers from older donors are at greater risk for ischemia-reperfusion injury [25-27], although there is no universal agreement on this point [28]. Transplantation within the MELD era was also associated with a greater likelihood of post-OLT renal recovery. We observed this in the entire cohort (Table 8) and in the subgroup requiring RRT at OLT only (Table 9). Advantages within the MELD era include improved surgical technique and peri-transplant management, both leading to improved allograft function [18,29-33]. We cannot, however, exclude other beneficial aspects of the MELD era, such as minimization of nephrotoxic immunosuppression and earlier transplantation of patients with pre-OLT renal dysfunction, especially those requiring RRT [34-36].

Third, most of the remaining predictors of renal recovery relate either to the severity of the recipient's liver disease or to the choice of immunosuppressive regimen post-OLT. Thus, within our entire cohort (Table 8), or within one or more subgroups (Table 9), independent predictors of renal recovery included a lower recipient MELD score, a decreased recipient total bilirubin, and a decreased recipient ALT. A decreased recipient BMI was also an independent predictor of renal recovery, and may reflect the absence of ascites. Alternatively, an increased recipient BMI may reflect obesity rather than ascites. In this case, renal recovery may be impaired because of obesity-associated inflammatory responses or an increased risk for a small-for-size allograft with resultant allograft dysfunction and renal hypoperfusion [37,38].

With respect to the immunosuppressive regimen, lack of use of cyclosporine or tacrolimus emerged as an independent predictor of renal recovery. This is most likely because of renal hypoperfusion and other potentially nephrotoxic effects associated with use of calcineurin inhibitors [39]. Patients not treated with a calcineurin inhibitor tended to receive sirolimus. Thus, of 434 patients who did not receive a calcineurin inhibitor, 417 (96%) received sirolimus; correspondingly, of 1286 patients who received a calcineurin inhibitor, only 27 (2%) received sirolimus (p=0.057).

One of the more intriguing findings of our study is the possibly beneficial effect of anti-thymocyte globulin induction, which emerged as independent predictor of renal recovery within our entire cohort (Table 8) and among patients requiring RRT at the time of OLT (Table 9). The most obvious benefit of anti-thymocyte globulin induction lies in the fact that its use permits minimization of the dose of calcineurin inhibitors [40]. However, there may be additional more direct benefits, as suggested by the fact that failure to use calcineurin inhibitors and administration of anti-thymocyte globulin induction were retained as independent predictors in our multivariate analysis. In addition to the prominent T cell-depleting effects of anti-thymocyte globulin, this polyclonal antibody binds to and neutralizes multiple antigens present not only on T cells but also on neutrophils and other inflammatory cells, particularly adhesion molecules and chemokine receptors, both of which are involved in the recruitment of inflammatory cells to areas of ischemia-reperfusion injury [40]. Finally, anti-thymocyte globulin may have increased the likelihood of renal recovery by minimizing the occurrence of acute allograft rejection. Several facts argue against this. The incidence of acute allograft rejection was very low overall, and not different in patients who received anti-thymocyte globulin versus those who did not anti- (0% vs. 0.8%, p=1.00). Moreover, on univariate analysis, the absence of an acute rejection episode failed to predict renal recovery (p = 0.77).

Finally, in contrast to some studies [4,13-15], our data suggest that among patients with pre-OLT renal dysfunction a shorter duration of time on the transplant list is not an independent predictor of post-OLT renal recovery. Indeed, for the entire cohort, a longer, and not a shorter, wait time predicted renal recovery (Table 8). Notably, when we restricted our analysis to patients who required RRT at both registration and OLT, and were therefore presumably dialysis-dependent, we observed the following. Neither the mean wait time (Table 10) nor the percentage of patients with wait times >8 weeks (Table 11) was statistically different for recipients recovering renal function vs. those lacking renal recovery (Table 10). Moreover, the percentage of dialysis-dependent patients with post-OLT recovery of renal function was virtually the same in recipients with wait times greater than 8 weeks vs. less than 8 weeks (46% vs. 45%). These data highlight the importance of the cirrhotic milieu in compromising renal function, and suggest that reversible prerenal factors may predominate for prolonged periods of times, even lengths of time typically thought to be indicative of chronic irreversible renal damage.

With respect to the length of time on RRT, several considerations may bear on the discrepancy between our results and those of previous studies. Patients with longer wait times may be healthier and able to survive longer until receiving an OLT. In addition, since the need for RRT was recorded only at registration and transplantation, we cannot be sure that dialysis-dependent patients required RRT for the entire intervening period. This point requires further study. In support of our findings, Machicao et al. reported a negative association between an elevated SCr pre-OLT and the development of progressive renal dysfunction post-OLT, suggesting recovery of renal function in these patients post-OLT [36]. In contrast, Bahirwani et al. found that the duration of renal dysfunction and the presence of pre-OLT diabetes mellitus were inversely correlated with post-OLT improvement and preservation of renal function [13]. Others studies have also suggested that a longer duration of pre-OLT renal dysfunction is a risk factor for non-recovery of renal function [6,14]. It is pertinent to note that some studies in which native kidney function was evaluated following OLT have observed recovery of native kidney function after protracted periods of time, between 75 and 365 days [6,17,41].

Our study has a number of strengths. The most important is our use of the UNOS database, containing prospective data from over 100 centers over a period of >20 years. An additional strength is the large size of our cohort, which is greater than that of previous studies [13,14]. In addition, we used a rigorous definition of recovery of renal function, requiring a composite endpoint of survival ≥29 days plus a SCr ≤1.5 mg/dL at discharge. Finally, we evaluated predictors of renal recovery not only in the entire cohort, but also in several subgroups of patients with varying degrees of pre-OLT renal dysfunction.

We note several limitations to our study. First, the retrospective nature of our study limited our ability to acquire data, for example, the requirement for intra-operative transfusion or vasopressors. Second, the UNOS database contains information only at specified time points, so we were unable to determine the exact duration of pre-OLT renal dysfunction. Third, we lacked information on the etiology of pre-OLT renal dysfunction. The distinction between pre-renal and renal causes is especially critical in patients with liver failure. Post-OLT recovery of renal function is more likely for patients whose renal dysfunction is attributable to reversible pre-renal factors from the cirrhotic milieu, and less likely for patients whose renal dysfunction results from intrinsic factors attributable to chronic irreversible damage. Finally, we used the MDRD formula to determine eGFR. While estimates of renal function in cirrhotic patients based on SCr are known to overestimate the true GFR, it is important to emphasize that our analysis was limited to the correlation between renal recovery and changes, rather than absolute levels, of eGFR.

## Conclusion

In summary, our study suggests that the most important independent predictor of renal recovery in OLT recipients is the absence of hepatic allograft dysfunction. The immunosuppressive regimen appears also to be important, with avoidance of calcineurin inhibitors and use of anti-thymocyte globulin induction both associated with post-OLT recovery of renal function. Importantly, the mechanism of action of anti-thymocyte globulin induction appears to be distinct from that of calcineurin inhibitor sparing. Our study further suggests that reversible prerenal factors attributable to the cirrhotic milieu play a major etiologic role in approximately 50% of patients with re-OLT renal dysfunction. Our data also indicate that the duration of renal dysfunction, even among those requiring RRT, is a poor way to discriminate reversible from irreversible renal dysfunction. Given the scarcity of organs available for SLKT, a clear need exists for additional studies, incorporating renal biopsy data. These studies will help not only to determine which patients should receive SLKT rather than OLT alone, but also to identify peritransplant regimens that enhance the likelihood of post-OLT renal recovery.

## Competing interests

The authors declare that they have no competing interests. No external funding supported this research.

## Authors’ contributions

JII is the Principal Investigator, and participated in study design, statistical analysis, manuscript creation and preparation, data management, and review of the literature. EF: participated in study design, review of the literature, and copy editing of the manuscript. SM participated in study design and review of the literature. JMD participated in study design, review of the literature, and statistical analysis. JSL is the Senior investigator, and participated in study design, statistical analysis, manuscript editing and preparation, and review of the literature. All authors read and approved the final manuscript and its revisions.

## Pre-publication history

The pre-publication history for this paper can be accessed here:

http://www.biomedcentral.com/1471-2369/14/147/prepub
